# The Role of Early Vitrectomy in the Healing of Retinal Lesions in Progressive Outer Retinal Necrosis

**DOI:** 10.1155/2022/7636052

**Published:** 2022-02-27

**Authors:** Hamid Riazi-Esfahani, Arash Mirzaei, Masoud Mirghorbani, Fariba Ghassemi, Mohammad Zarei, Elias Khalili Pour, Nazanin Ebrahimiadib

**Affiliations:** Retina Service, Farabi Eye Hospital, Tehran University of Medical Sciences, Tehran, Iran

## Abstract

**Purpose:**

To report on the efficacy of early pars plana vitrectomy (PPV), silicone oil (SO) tamponade, and intravitreal ganciclovir injection in the treatment of a case with progressive outer retinal necrosis (PORN). *Case Presentation*. A 33-year-old man with a history of shingles on the chest skin 2.5 months ago presented with progressive vision loss in both eyes over the past 20 days. Fundus examination revealed retinal necrosis with perivascular clearance. Human immunodeficiency virus (HIV) infection was confirmed by western blot analysis. Treatment with intravenous acyclovir and intravitreal ganciclovir injections was unable to stop the progression of retinitis. Along with highly active antiretroviral therapy, the patient underwent PPV with SO tamponade and intravitreal ganciclovir injection in both eyes. A few days after surgery, retinal lesions started to improve.

**Conclusion:**

Early PPV, SO tamponade, and intravitreal ganciclovir injection may be considered an effective intervention in PORN patients with an unfavorable response to medical treatment.

## 1. Introduction

Progressive outer retinal necrosis (PORN) syndrome was first identified in 1990 in two patients with acquired immunodeficiency syndrome (AIDS) [1]. It is distinguished from acute retinal necrosis by its rapid progression and vision loss caused by early posterior pole involvement, absence of vitreous reactivity, and relative sparing of the retinal vascular system. Varicella-zoster virus (VZV) infection is the most common cause [2].

This condition is characterised by a poor response to standard antiviral medications, and the visual prognosis remains guarded [2]. Final visual acuity has been reported to be no light perception (NLP) in up to 67 percent of patients with PORN [3]. Due to rapid progression and ominous outcomes, empiric therapy usually begins before a definitive diagnosis.

Herein, we report a case of PORN syndrome with an initial unfavorable response to medical treatment and subsequent successful management with early pars plana vitrectomy (PPV), silicone oil (SO) tamponade, and intravitreal ganciclovir injection.

## 2. Case Report

A 33-year-old man presented to the retina clinic with progressive vision loss in both eyes over the past 20 days. The best-corrected visual acuity (BCVA) was 20/40 in both eyes. He reported an unintentional weight loss of 17 kg over the past two years. His medical history was notable for a zoster infection involving his chest dermatome as well as oral candidiasis 2.5 months prior to presentation.

**Figure 1:**
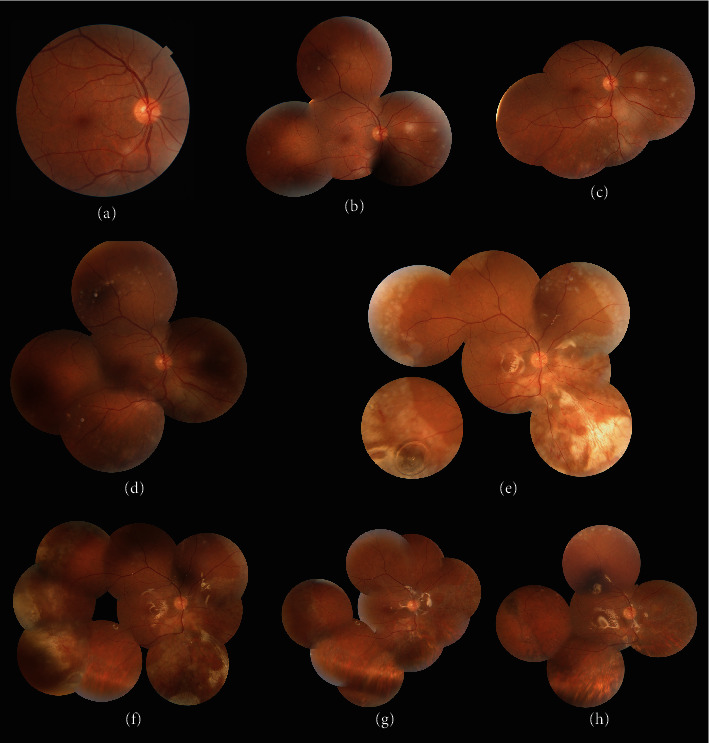
(a) Fundus photograph of the right eye (OD) at presentation shows white necrotic patches in the vicinity of the inferior arcade. (b) Montage fundus photograph of OD on day 4: retinal lesions progressed, intravenous acyclovir dosage was increased to 850 mg three times per day, and the second dose of intravitreal ganciclovir (2 mg/0.1 cc) was administered. (c) Three days later, despite medical treatment, the lesions continued to progress. (d) On day 10, lesions coalesced and showed an increase in size. (e) One day after pars plana vitrectomy with silicone oil tamponade and prophylactic endolaser. (f) One week after the right eye surgery (day 20). (g) One month after the right eye vitrectomy, the lesions regressed significantly. (h) Seven months after initial presentation, lesions near completely healed.

**Figure 2:**
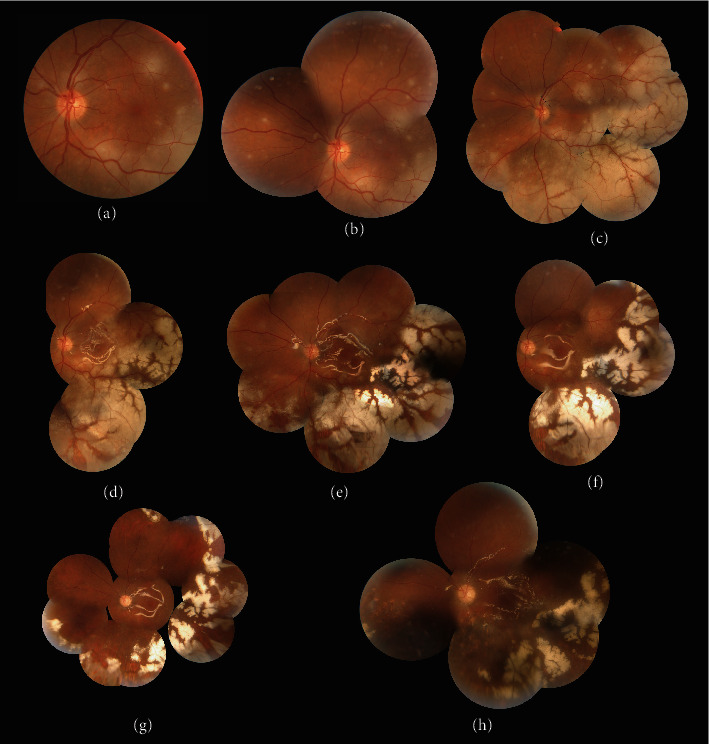
(a) Fundus photograph of the left eye (OS) at presentation shows white necrotic retinitis patches encroaching the macula from the inferior and temporal area. Scattered hemorrhages are evident along the inferior arcade. There is no significant vitritis. (b) Montage fundus photograph of OS on day 4: retinal lesions progressed, the intravenous acyclovir dosage was increased to 850 mg three times per day, and the second dose of intravitreal ganciclovir (2 mg/0.1 cc) was administered. (c) Three days later, despite medical treatment, the lesions coalesced and showed an increase in size. Perivascular sparing can be appreciated. (d) One day after pars plana vitrectomy with silicone oil tamponade and intravitreal ganciclovir injection. (e) On day three postoperatively, lesions regressed, especially from the macular area. (f) One week after the left eye surgery (day 20). (g) One month after vitrectomy, the lesions resolved obviously and got sharper borders. (h) Seven months after initial presentation, the lesions regressed significantly.

Slit-lamp examination of external adnexal and anterior segment revealed unremarkable findings in both eyes. Funduscopy of the right eye showed round white patches distributed across the posterior fundus, becoming denser in the inferonasal quadrant. There were no obvious vascular involvement and vitreous inflammation. (Figure 1(a)). Similar retinal lesions with more extensive involvement were observed in the left eye. The patches coalesced in the area posterior to the equator, particularly in the inferior quadrants. Some small dot hemorrhages were noticed, especially along the inferior arcade. No vitreous inflammation was observed. (Figure 2(a)).

On admission, intravenous (IV) acyclovir 700 mg (10 mg/kg), three times per day, and oral aspirin 80 mg daily were commenced. On day 1, the patient underwent intravitreal injection of ganciclovir in both eyes (2 mg/0.1 cc). Polymerase chain reaction (PCR) of the aqueous sample was positive for Varicella zoster virus (VZV) and Human Herpes Virus 6 (HHV6). Additionally, positive serum human immunodeficiency virus (HIV) antibodies were detected using an initial enzyme-linked immunosorbent assay (ELISA) method and validated using a western blot test. At the time of admission, the CD4 count was 42 cells/mm3.

On day 4, retinal lesions continued to progress in both eyes. The intravenous acyclovir dose was increased to 850 mg three times per day, and both eyes received a second dose of intravitreal ganciclovir (2 mg/0.1 cc) (Figures 1(b) and 2(b)). The patient first attempted to deny HIV diagnosis and resisted the initiation of highly active antiretroviral therapy (HAART), but after a few days, he participated for treatment with the help of psychiatric counselling. Finally on day 7, the patient was started on HAART.

Three days later, despite medical treatment, the lesions expanded with sparing of perivascular areas and the BCVA of the left eye dropped to the counting fingers (CF) at 1 meter (Figures 1(c) and 2(c)). On day 10, the patient underwent 23-gauge PPV and SO tamponade along with intravitreal ganciclovir (2 mg/0.1 cc) injection in the left eye (Figure 2-D). Three days following surgery (day 13), the lesions in the left eye began to heal, while the lesions in the right eye continued to enlarge, lowering the BCVA to 20/200 (Figures 2(e) and 1(d). The same surgical procedure was scheduled for the right eye (Figure 1(e)). Barrier endolaser photocoagulation along the posterior border of the retinal lesions was performed in the right eye in contrast to the left eye (Figures 1(e) and 2(e)). Due to the proximity of the necrosis to the macula, posterior vitreous detachment (PVD) was not induced during the vitrectomy of the left eye, and the vitreous was shaved over the necrotic regions and towards the vitreous base using a very low aspiration mode. The posterior hyaloid was separated from the optic disc up to the equator using triamcinolone for better visualization of vitreous during the vitrectomy of the right eye, and the remaining vitreous was meticulously shaved using the shaving mode of the vitrectomy instrument.

After two weeks of IV acyclovir, the patient was switched to oral valacyclovir 1 g three times daily. On day 20, the BCVA improved to 20/60 in the right eye (OD) and 20/200 in the left eye (OS) (Figures 1(f) and 2(f)).

One month after PPV, the lesions of both eyes improved dramatically. The BCVA was stabilized at 20/60 OD and 20/200 OS (Figures 1(g) and 2(g)). After the posterior lesions in the left eye were resolved, prophylactic barrier laser photocoagulation was performed on the areas away from the fovea. On day 60, the CD4 count had increased to 124 cells/mm^3^.

Seven months after presentation, all lesions significantly regressed and the BCVA was 20/60 OD and 20/100 OS (Figures 1(h) and 2(h)). As the patient's CD4 count remained subnormal at 147 cells/mm3, valacyclovir 1 g three times daily was continued in addition to HAART.

## 3. Discussion

PORN is a potentially blinding disease that often responds poorly to currently available antiviral therapies. This condition is most frequently found in immunocompromised patients with a shingles history [1–3]. Due to the disease's rarity, there has been no prospective, randomised clinical trial; hence, management recommendations are based on case reports/series.

The patient did not respond to the initial intravenous acyclovir treatment or the subsequent intravitreal ganciclovir injection in this report. We had limited access to more potent antivirals such as Foscarnet, as a result, we used PPV in conjunction with SO tamponade and intravitreal ganciclovir injection. Within two days of surgery, this approach resulted in a dramatic improvement of the lesions, which lasted for seven months.

Mehrotra et al. recently described a case of PORN in a 43-year-old male who was originally treated with intravenous acyclovir and intravitreal ganciclovir, followed by oral valacyclovir. One month later, the patient had retinal detachment. After one month, PPV with SO tamponade and intravitreal and intravenous ganciclovir injection resulted in retinal reattachment and an improvement in visual acuity to 20/200 [4].

To the best of our knowledge, this is the first report of an early vitrectomy for a PORN syndrome patient. We propose two possible explanations for the effectiveness of simultaneous SO tamponade and intravitreal ganciclovir injection. The first possibility is that the injected ganciclovir concentration is greater in the “subsilicone space,” which is in close proximity to the retinal lesions. The other possibility is that SO has an antiviral effect, as demonstrated in an in vitro investigation against HSV-1 [5]. However, there is no data of SO antiviral effect against VZV or CMV in the literature.

Although the findings of a recent meta-analysis in patients with acute retinal necrosis support early vitrectomy in lowering the rate of retinal detachment as a potentially devastating complication, more research is needed to determine the efficacy of early vitrectomy in individuals with PORN syndrome in terms of controlling retinal inflammation and preventing retinal detachment [6].

While the patient has a promising BCVA with attached retina in both eyes seven months after PPV and SO tamponade, the patient's final outcome, particularly after SO removal, requires long-term follow-ups.

Finally, our limited experience, in this case, illustrates that for progressive PORN patients despite conventional antiviral regimens or in situations with limited access to more potent antivirals, early vitrectomy and silicone oil tamponade with concomitant intravitreal ganciclovir injection can show short-term efficacy.

## Data Availability

The datasets used in the current study are available upon reasonable request.
